# Medical and Philosophical News

**Published:** 1781-12

**Authors:** 


### SECTION III.

### Medical and Philosophical News.

THE Royal Medical Society at Paris have propofed the following queftion for a prize of 600 livres: "What are the figns that "announce a difpofition to Phthifis Pulmonalis, "and what are the means of preventing the "attack, or ftopping the progrefs of that dif-

" eafe ?" The differtations on this fubject are to be written in Latin or French, and fent to M. Vicq. D'Azyr, Secretary to the Society, before the 1ft of January 1783.

---

The Royal Academy of Sciences at Copenhagen have offered a gold medal of the value of 100 rix dollars to the perfon " who fhall " carry to a higher degree of perfection and ac- " curacy the means of afcertaining the falubrity " of the air, and who fhall confirm the truth " of his precepts by new experiments." The differtations on this fubject are to be written in Latin, Danifh, French, or German, and fent before the 31ft of Auguft 1782 to M. de Luxdorph, Knight of the Order of Danebrog and Prefident of the Society.

---

The Abbé Mann, of the Academy of Sciences at Bruffels, after having been long fubject to the gout, began about three years ago to take the *Extractum Cicutæ*, in the ufe of which he has perfevered ever fince, and for the laft eighteen months has had no return of his complaint. We are informed that he means foon to publifh an account of his cafe.

---

Mr.

Mr. Rinman, an ingenious Swedish chemist, has invented a method of lining copper and other culinary utensils with an enamel composed of equal parts of fusible spar and gypsum, calcined and powdered. After moistening the surface of the metal this composition is directed to be spread over it, and then the vessel is to be exposed to a certain degree of heat.

---

On Monday, July the 9th 1781, Dr. Morizot des Landes, physician at Paris, was called to the assistance of the Jacobin Monks in that city. These persons, who were twenty-one in number, complained of violent head-ach, pain of the bowels, diarrhœa, excessive weakness of the whole body, particularly of the legs, and a constant pain at the fore part of the thighs. All of them had more or less of fever, and some were troubled with cramp in the calves of their legs. They who were first attacked had likewise experienced acute pain at the stomach, and anxiety about the præcordia.

So many persons complaining at the same time of the same symptoms left no room to doubt that a common cause had acted on them all. It appeared that on the preceding Friday and Saturday their dinners had consisted of fish dressed on Thursday evening in a copper saucepan, and that the cook after drawing off the water had poured vinegar on the fish, which in this state had been suffered to remain for a considerable time in the vessel. Some of the monks began to be affected on the Friday, the rest not till the day following. From this account it appeared clear that their complaints originated from the poison of copper.

The remedies employed by Dr. des Landes consisted of mild purges, glysters, broths, and diluting, mucilaginous liquors. By means of these all the monks were perfectly recovered in five or six days, but a visitor, who dined at the convent on the Saturday, and who took an emetic, continued in a very ill state of health till the month of September.—*Journ. de Med.*

---

M. Dombay, the botanist mentioned in a former part * of this volume, has sent to his friend M. de la Lande at Paris, a specimen of *Quinoa* seed, which is said to be equal to rice in

* Page 274.

goodgoodneſs. It is produced by a ſpecies of *chenopodium* that grows on the mountains of Peru, and might probably be cultivated to advantage in Europe. Each plant produces about a thouſand ſeeds.

M. Dombay has likewiſe ſent home two preparations of potatoes, one called by the Peruvians *pape ſeca* and the other *Chuno*, from which thoſe people derive a conſiderable part of their nouriſhment.

The *pape ſeca* is prepared by boiling the potatoes in water, after which they are peeled, and expoſed to the ſun and air till they are perfectly dry.

The proceſs for making the *Chuno* conſiſts in freezing the potatoes, and depriving them of their peel, after which they are placed in a current of water during fifteen or twenty days, and then dried in the ſun.

---

Extract of a letter from Dennis Ryan, M. D. to Dr. Simmons, F. R. S. dated at Bath in Jamaica, April 24th 1781.

" During a month's reſidence at this place I have carefully noticed the ſtate of the atmo-

ſphere.

ſphere. I ſhall not trouble you w'th the whole of my journal, but ſhall content myſelf with giving you the greateſt and leaſt heights of the thermometer in the ſhade. My obſervations have generally been made three times a day; viz. at ſeven o'clock in the morning, at noon, and at ten at night.

| | | | Greateſt Height. |
|---|---|---|---|
| Morning, | *April* 15, 21, and 23, | — | 78° |
| Noon, | —— 12, 20, | — | 84 |
| Night, | —— 17, 18, | — | 79 |
| | | | Leaſt Height. |
| Morning, | *April* 2 and 10, | — | 70° |
| Noon, | —— 22 and 23, | — | 79 |
| Night, | —— 11, | — | 74 |

In order to aſcertain the heat of the Bath water, I immerſed a thermometer into a calibaſh full of it, and in the ſpace of about two minutes the mercury roſe to 122°. I then held it about a minute under the ſpout as it comes down from the rock, and the quickſilver pointed at 123°, which, if I miſtake not, is by ſome degrees greater than the heat of the Bath water in England.

The water at its ſource emits a ſtrong ſulphureous vapour, which affects a perſon at the diſtance of ſeveral yards. It is very ſparkling in in the glaſs, and in its taſte reſembles the English Bath water, but as yet there has been no ſatisfactory analyſis of it. As an external application it is found to do good in obſtinate ulcers, and in gouty and rheumatic pains. Taken internally it affords great relief in bilious complaints, and in obſtructions of the abdominal viſcera, but if adminiſtered without the previous uſe of ſome laxative medicines, it is apt to bring on great heat, head-ach, and other diſagreeable ſymptoms."

---

At the anniverſary meeting of the Royal Society of London, on the 30th of November 1781, Mr. William Herſchel of Bath received the Copleian medal from the hands of the Preſident, as a reward for his having diſcovered a new ſtar.

---

A new work by Sir Clifton Wintringham, Bart. entitled *Adverſaria Medica*, is now in the preſs.

---

Some experiments have lately been made in the Royal Infirmary at Edinburgh with the *digi-*

*digitalis purpurea*, or *fox-glove*, which prove it to be a medicine of considerable efficacy in dropsy.

